# Plexin C1 influences immune response to intracellular LPS and survival in murine sepsis

**DOI:** 10.1186/s12929-024-01074-x

**Published:** 2024-08-21

**Authors:** Alice Bernard, Claudia Eggstein, Linyan Tang, Marius Keller, Andreas Körner, Valbona Mirakaj, Peter Rosenberger

**Affiliations:** Department of Anaesthesiology and Intensive Care Medicine, Eberhard-Karls University Tübingen, Hoppe-Seyler-Straße 3, 72076 Tübingen, Germany

**Keywords:** Noncanonical inflammasome, Caspase-11, Sepsis, Plexin C1

## Abstract

**Background:**

Intracellular sensing of lipopolysaccharide (LPS) is essential for the immune response against gram-negative bacteria and results in activation of caspase-11 and pyroptotic cell death with fatal consequences in sepsis. We found the neuronal guidance receptor plexin C1 (PLXNC1) influences the intracellular response to LPS.

**Methods:**

We employed a murine model of sepsis via cecal ligation and binding (CLP), using PLXNC1-/- mice and littermate controls, and additionally transfected murine bone-marrow-derived macrophages (BMDMs) from both genotypes with LPS to achieve activation of the noncanonical inflammasome ex vivo. Additionally, we transfected the PLXNC1 ligand SL4c-d in vivo and ex vivo to examine its effect on intracellular LPS response.

**Results:**

We found the neuronal guidance receptor PLXNC1 dampens the intracellular response to LPS by interacting with adenylate cyclase 4 (ADCY4) and protein kinase A activity, which in turn diminishes caspase-11 expression. The absence of PLXNC1 results in excessive inflammation marked by increased cytokine release, increased secondary organ injury and reduced sepsis survival in a murine sepsis model induced by CLP. Notably, administration of SL4c-d—peptide ligand of PLXNC1—reduces the inflammatory response during CLP-induced sepsis and improves survival.

**Conclusions:**

These results elucidate a previously unknown mechanism for PLXNC1 suppressing excessive noncanonical inflammasome activity and offer a new potential target for treatment of sepsis with its detrimental effects.

**Supplementary Information:**

The online version contains supplementary material available at 10.1186/s12929-024-01074-x.

## Introduction

With over 30 million cases worldwide and a mortality rate of up to 50%, the burden of sepsis on the healthcare systems remains enormous [1]. Beyond that, sepsis survivors often experience long-term damage, such as cognitive impairment or cardiovascular complications [2, 3].

Gram-negative bacteria are a common cause of sepsis, and lipopolysaccharide (LPS) derived from these pathogens potently triggers inflammation through intracellular stimulation of the noncanonical inflammasome [4]. Cytoplasmatic LPS binds to the caspase-recruitment domain (CARD) of caspase-11 (in mice) and caspase-4 or caspase-5 (in humans). Caspase-11 is then activated by proximity-induced oligomerization through LPS [5], resulting in secretion of the proinflammatory cytokines IL-1ß and IL-18 and induction of pyroptotic cell death mediated by gasdermin D (GSDMD) [6]. Evidence points to caspase-11 being an important effector in acute inflammation in vivo [7]. While one earlier study found caspase-11 to be of no significance during polymicrobial sepsis induced by cecal ligation and puncture (CLP) [8]—a common model of sepsis—, later authors report of a significant survival advantage for mice with a depletion of caspase-11 [9, 10]. Furthermore, the absence of caspase-11 reduced organ damage during both polymicrobial sepsis [11] and LPS-induced systemic inflammation [12]. Therefore, caspase-11 is a promising therapeutic target for sepsis, but regulation of its activity during sepsis-induced immune response is not well understood.

Recent research showed that neuronal guidance proteins influence the immune response [13, 14]. Plexins—a group of neuronal guidance receptors—are known as important “stop” mechanisms during the movement of cells and progression of malignant disease [15]. The neuronal guidance receptor plexin C1 (PLXNC1) belongs to the plexin receptor superfamily and is expressed on various immune cells such as neutrophils, dendritic cells, B cells and T cells [16]. So far, the receptor has been found to influence sterile models of inflammation, e.g. peritonitis induced by Zymosan A [17], hepatic ischemia–reperfusion injury [18], and ventilator-induced lung injury [19].

Binding of its ligand Semaphorin 7A (Sema7A) to PLXNC1 promotes transmigration of neutrophiles in vitro and leads to the formation of platelet-neutrophil complexes during lung injury [20], pointing at a beneficial effect of the receptor in this sterile inflammatory context. Sema7A has also been shown to have a positive influence on the resolution of inflammation [14], and its core protein Sema 7A loop 4c-d (SL4c-d) contains the binding side for the receptor [21]. In previous models, application of SL4c-d was able to reverse the effect of the PLXNC1 knock-out [17, 19].

The aim of this study was to elucidate a possible stop mechanism through PLXNC1 in the control of intracellular response to LPS. In addition, we aimed to identify a therapeutic potential of PLXNC1 during sepsis.

## Material and methods

### Mice

*PLXNC1*^-/-^ mice—originally provided by Amgen (Amgen, Thousand Oaks, California, USA)—were established on a C57BL/6 background and bred in our local animal facilities at the University of Tübingen, Germany. These *PLXNC1*^-/-^ mice show a stable deletion of PLXNC1-expression in all body tissues and have been used researching the receptor by various researchers for over a decade [16, 18, 22]. C57BL/6 mice provided by Charles River (Charles River, Sulzfeld, Germany) were backcrossed with *PLXNC1*^-/-^ mice to obtain a wild-type genotype as littermate controls.

### In vivo mouse models

Animal experiments were performed in accordance with the German Animal Welfare Law and approved by the appropriate local authorities (i.e. Regierungspräsidium Tübingen). Polymicrobial sepsis was induced in respective mice by 50% ligation of the caecum and puncture using a 20 gauges needle. For sham surgery, the cecum was carefully removed and replaced without ligation or puncture.

The model of *E. coli*-induced sepsis was established by intraperitoneal injection of viable bacteria (5 × 10^7^ CFUs/mouse; ATCC, #19138, Manassas, Virginia, USA).

In corresponding experiments, animals were euthanized 24 h after CLP. Peritoneal lavage fluid, blood, and organ tissue samples were retrieved for further analysis.

### Histological assessment

Liver and colon tissue samples were retrieved from mice after indicated experiments. Samples were fixed with 4% formalin overnight after harvesting, and embedded in paraffin and stained with H&E. A Leica DM5000B microscope (Leica Microsystems, Wetzlar, Germany) was used to acquire images of tissue sections. Liver damage was assessed based on the Suzuki score [23], and intestinal damage was assessed based on Chiu’s score [24] by two independent, blinded examiners.

### Cytokine measurement and LDH assay

Concentrations of cytokines (IL-6, TNF-α, IL-1ß, and keratinocyte-derived chemokine [KC]) in peritoneal lavage and cell culture supernatants were determined with ready-to-use murine sandwich ELISAs (R&D Systems, Minneapolis, Minnesota, USA). LDH activity was measured in cell culture supernatants using a CytoTox 96© Non-Radioactive Cytotoxicity Assay Kit according to the manufacturer's instructions (Promega, Madison, Wisconsin, USA). IL-18 concentration was measured using a murine IL-18 ELISA kit (MBL Life Science, #7625, Japan) according to the manufacturer’s instructions. ELISA plates were read in an Infinite® M200 Pro Plate Reader (Tecan, Männedorf, Switzerland).

### Caspase-11 measurement

Caspase-11 concentration in peritoneal lavage fluid and cell culture supernatants was measured using a commercially available ELISA kit (Abbexa Ltd., abx255239, Cambridge, UK) in accordance with the manufacturer’s instructions.

### Protein isolation and Western blot analysis

Peritoneal lavage fluid was harvested 24 h after CLP and centrifuged at 2000 rpm to precipitate the cellular fraction. The cellular fraction was then reconstituted in ice-cold acetone and methanol. In cell culture experiments, cells were lysed in RIPA buffer with protease inhibitor (1:100). Proteins were separated via SDS‒PAGE and transferred to a 0.2 µm PVDF membrane for analysis of caspase-11 (Novus Bio, #14D9, Littleton, Colorado, USA), caspase-1 (AdipoGen, #mAB Casper-1, Rockland, Maine, USA), ASC (LSBio, #LS-C413166, St. Louis, USA), GSDMD (Abcam, #ERP19828, Cambridge, UK), NLRP3 (LSBio, LS-C148764, St. Louis, Missouri, USA). Species-matched alkaline phosphatase-conjugated secondary antibodies were used. Protein detection was performed using a BCIP/NBT substrate or visualized by enhanced chemoluminescence (Amersham Pharmacia Biotech, Amersham, UK).

GAPDH expression was determined using an anti-GAPDH antibody (Sigma‒Aldrich, #G9545, St. Louis, Missouri, USA). Labeled protein bands were visualized by enhanced chemoluminescence.

### Isolation of BMDMs

BMDMs were obtained from femurs and tibias of mice after euthanasia. Bones were washed out with PBS using a 27 gauges needle, and then cultured in a 10 cm Petri dish at 37 °C for a minimum of 6 days in RPMI 1640 medium (Sigma‒Aldrich, St. Louis, USA) supplemented with 10 ng/ml murine GM-CSF (R&D Systems, 415-ML, Minneapolis, Minnesota, USA). For stimulation in culture, cells were plated in a 48-well plate at a density of 5 × 10^5^ cells/well.

### Isolation and differentiation of human peripheral blood monocytes/macrophages

Human peripheral blood cells (PBMCs) were isolated from healthy donors at the Blood Bank of Eberhard Karls University of Tübingen by gradient centrifugation using Histopaque-1077 (MilliporeSigma, Burlington, Vermont, USA). In short, 20 ml of blood were mixed with PBS at a 1:1 ratio, and carefully layered onto 15 ml of Histopaque-1077 before being centrifuged (30 min, 1500 U/min, 20 °C). PBMCs—gathered in an opaque phase between erythrocytes at the bottom and the plasma phase above—were collected and cultured in RPMI 1640 medium supplemented with 10 ng/ml human recombinant GM-CSF (Miltenyi Biotec, #130–093-866, Bergisch Gladbach, Germany) at 37 °C in a 5% CO_2_ atmosphere for a minimum of 6 days for differentiation into monocytes/macrophages.

### Stimulation of noncanonical inflammasome (caspase-11)

BMDMs from *PLXNC1*^-/-^ and wild-type control mice were primed overnight with 50 ng/ml LPS from *E. coli* subtype O111:B4 (Sigma‒Aldrich, L4392-1MG, St. Louis, Missouri, USA) to induce upregulation of caspase-11, as well as of pro-IL-1β, and pro-IL-18 [5, 25]. Samples were then transfected with the same LPS for actual caspase-11 activation at a total concentration of 2 µg/ml LPS in the presence of Lipofectamine 2000© (3 µl/ml; Thermo Fisher, #11668019, Waltham, Massachusetts, USA) according to the manufacturer's instructions [25]. After 6 h, supernatants were collected for LDH activity and cytokine level measurements, and cells were lysed in RIPA buffer for protein extraction.

### Digitonin assay

BMDMs from C57BL/6 wild-type mice were prepared and transfected with LPS in a 12-well plate as described above. After stimulation, cells were detached with Accutase© Cell Detachment Solution (InvivoGen, #00-4555-56, San Diego, California, USA) and pelleted by centrifugation. Resulting cell pellets were resuspended in 60 µl of digitonin buffer (Sigma‒Aldrich, #D141, St. Louis, Missouri, USA) before being centrifuged for separation of the cytosolic fraction and the membrane fraction. The supernatant constituting the cytosolic fraction was resuspended in 5 × Laemmli buffer; the pellet containing the membrane fraction was resuspended in PBS before adding 5 × Laemmli buffer. Proteins were then separated via SDS‒PAGE and transferred onto a 0.2 µm PVDF membrane. Plexin C1 expression was visualized using a polyclonal anti-PLXNC1 antibody (R&D, #AF5375, Minneapolis, Minnestoa, USA) and a BCIP/NBT substrate.

### Transfection of SL4c-d ex vivo and in vivo

SL4c-d was custom-produced by ProImmune Ltd. (Oxford, UK) based on the following amino acid sequence: BSACRGDQGGESSLSVSKWNTF. For ex vivo experiments, 3 µg/ml SL4c-d was transfected into BMDMs from C57BL/6 wild-type mice using an Xfect™ Protein Transfection Kit (Takara, Kusatsu, Japan) according to the manufacturer’s instructions. Two hours after SL4c-d transfection has been completed, media was changed, and cells were primed with LPS (50 ng/ml) overnight. To complete activation of the noncanonical inflammasome, cells have then been transfected with LPS as described above.

To achieve cellular internalization of SL4c-d in vivo, SL4c-d was coupled to the cell-penetrating peptide Arg9 (Genaxxon, P2286.9505, Ulm, Germany) at a 1:1 ratio. First, 5 µg of SL4c-d and 5 µg of Arg9 per animal were dissolved together in 500 µl of sterile PBS and incubated for 30 min; before i.v. injection, the total volume was adjusted to a maximum of 5 ml/kg using sodium chloride. Mice received one i.v. injection of SL4c-d/Arg9 or Arg9 into the tail vein, respectively, directly after CLP. For survival analysis, animals received one injection every 24 h [26, 27].

### Protein kinase A activity assay

PKA activity was measured using a colorimetric activity assay kit (Thermo Fisher, EIAPKA, Waltham, Massachusetts, USA) according to the manufacturer’s instructions.

### AST assay

Murine serum AST levels were determined using an Aspartate Aminotransferase Colorimetric Assay (Cayman Chemical, #701640, Ann Arbor, Michigan, USA) according to the manufacturer’s instructions.

### Next-generation sequencing

NGS of RNA was performed on murine BMDMs after LPS transfection as described above. RNA was isolated utilizing an RNeasy Micro Kit (QIAGEN, #74034, Hilden, Germany) according to the manufacturer’s protocol. Isolated RNA was processed by MFT Services and the Core Unit for Applied Genomics, Tübingen. For data analysis, raw gene expression data were filtered with the criterion of a minimum expression value of 1 counts per million (cpm) in at least three samples. The samples were analyzed with respect to their pairwise similarity. Spearman rank correlation and hierarchical clustering analyses were performed to determine relationships between values. Differential gene expression analysis was conducted based on a gene expression data set filtered with the abovementioned criterion. A statistical model incorporating pairwise relationships and group properties of samples was applied. Genes identified as up- or downregulated in the corresponding group according to the log fold change (logFC) value, p value, and false discovery rate (FDR) were imported into Ingenuity Pathway Analysis (IPA) software (QIAGEN, Hilden, Germany) to identify altered pathways and regulatory mechanisms. Log10 transcripts per kilobase million (TPM) values were used to produce detailed heatmaps including individual values for visualization.

### Coimmunoprecipitation

Co-IP was performed as described in the instruction manual of the Thermo Scientific™ Pierce™ Coimmunoprecipitation Kit (cat. number 24149).

Human peripheral blood monocytes/macrophages were cultured in a 10 cm Petri dish. Cells were lysed in 500 µl of lysis/wash buffer, mixed well by pipetting on ice for 5 min and incubated on a rotator at 4 °C for 30 min. After centrifugation at 13000×*g* for 10 min, the supernatant was transferred to a new tube, and the protein concentration was measured by processing with a Pierce™ BCA Protein Assay Kit (Thermo Fisher, #23225, Waltham, Massachusetts, USA) and analyzed in an Infinite® M200 Pro Plate Reader (Tecan, Männedorf, Switzerland). Preclearing of lysates using control agarose resin was performed as described in the instruction manual. For antibody immobilization, 10 μg of affinity-purified antibody specific for either Plexin C1 (Santa Cruz Biotechnology #sc390216, Santa Cruz, California, USA) or ADCY4 (Invitrogen, PA5-101283, Waltham, Massachusetts, USA) was prepared for binding by adjusting the volume to 200 μl. As an IgG control, 10 µg of mouse IgG (Dako, X0943, Glostrup, Denmark) or 10 µg of rabbit IgG (Thermo Fisher Scientific, #31235, Waltham, Massachusetts, USA) was used. For co-IP analyses, 1 mg of protein was diluted in 200 µl of IP lysis/wash buffer, added to the appropriate resin and incubated with rocking overnight at 4 °C. Prey proteins were eluted in 60 μl of elution buffer. Samples were analyzed by Western blotting via BCIP/NBT method as described above.

### Phospho explorer antibody array

Twenty-four hours after CLP, peritoneal lavage fluid was collected from *PLXNC1*^-/-^ animals and their littermate controls. Proteins were isolated and pooled in accordance with the instructions for the Phospho Explorer Antibody Array (FullMoon BioSystems, PEX100, Sunnyvale, California, USA). For each antibody, the average signal intensity of two replicates was normalized to the median signal of all antibodies in the array. The presented fold change represents the ratio of the signal in *PLXNC1*^-/-^ animals to the signal in their littermate controls. Data analysis was performed with the IPA software (QIAGEN). Pathways were verified and updated based on recent literature, the KEGG database (hsa04150, hsa04064, hsa04151) and the Reactome database (R-HSA-165159, R-HSA-5676590, R-HSA-198203). A schematic image picturing the respective pathway was constructed using biorender.com based on the data produced by the IPA software.

### Statistical analysis

Statistical analysis was performed using GraphPad Prism© 7.0 (GraphPad, San Diego, California, USA). Unpaired Student’s t test was applied for comparisons between two groups. The results are presented as the means ± SEMs. A *p *value < 0.05 was considered significant.

During survival experiments, animals were monitored for a maximum of 96 h; overall survival was examined by creation of a Kaplan-Meyer curve to estimate survival probability, and with a log-rank-test to analyze probable significance. As required by the German Animal Welfare Law and the respective German government bodies, a statistical analysis has been performed beforehand to determine the number of animals needed to proof or rule out our respective hypothesis.

## Results

### Plexin C1 reduces mortality and secondary organ injury caused by polymicrobial sepsis

**Fig. 1:**
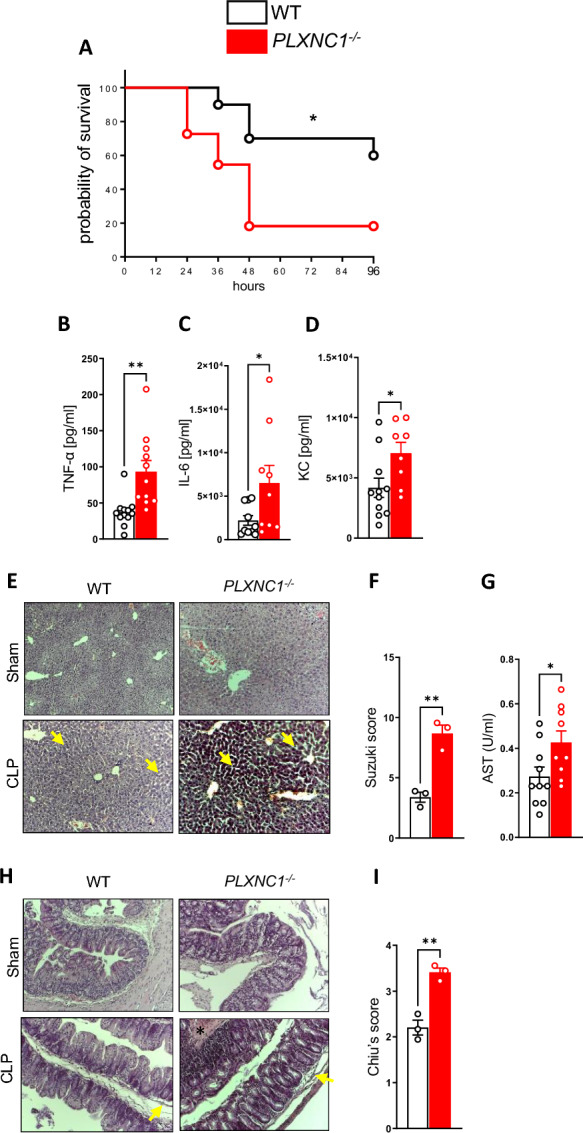
PLXNC1 reduces sepsis mortality and suppresses inflammatory damage in vivo. A model of midgrade polymicrobial sepsis using caecal ligation and puncture (CLP) was established in mice. **A** Survival curves of *PLXNC1*^-/-^ mice and their littermate controls over the course of 96 h. Levels of **B** TNF-α, **C** IL-6 and **D** KC measured by ELISA in the peritoneal lavage fluid of *PLXNC1*^-/-^ mice 24 h after CLP induction. **E** Histological analysis of liver tissue sections and **F** evaluation of liver damage by the Suzuki score (yellow arrows pointing at vacuolation and congestion of hepatocytes). **G** Serum levels of AST. **H** Histological analysis of colon sections (arrows pointing at epithelial lifting; asterisk marking necrosis), and evaluation of intestinal damage by **I** Chiu’s score. (The data are presented as the means ± SEMs; n = 5–11 mice/group; histology, n = 3 mice/group; *p < 0.05; **p < 0.01 and ***p < 0.001 as indicated)

To evaluate a potential influence of PLXNC1 on the inflammatory processes during sepsis, we subjected *PLXNC1*^-/-^ mice to polymicrobial sepsis via CLP. This showed a significant survival disadvantage for *PLXNC1*^-/-^ mice compared to littermate controls (Fig. 1A). Twenty-four hours after sepsis induction, levels of the proinflammatory cytokines TNF-α, IL-6, and KC (Fig. 1B–D) were significantly higher in the peritoneal lavage fluid of *PLXNC1*^-/-^ mice, suggesting an inflammation-suppressing function for PLXNC1 in vivo during sepsis.

Furthermore, depletion of PLXNC1 resulted in more severe secondary organ injury of liver and intestines: In Fig. 1E–G, we see markedly more vacuolation and congestion of hepatocytes (arrows) in *PLXNC1*-/- (Fig. 1E), and a higher degree of liver damage, as assessed by Suzuki score for hepatic injury (Fig. 1F), as well as higher AST levels (Fig. 1G). Intestinal injury is also more severe in *PLXNC1*-/- mice, with a higher degree of epithelial lifting (arrows), disrupted crypt architecture as well necrosis (asterisk) and bleeding (Fig. 1H), as assessed by Chiu’s score (Fig. 1I).

### Plexin C1 changes the expression of hallmark inflammasome proteins

**Fig. 2:**
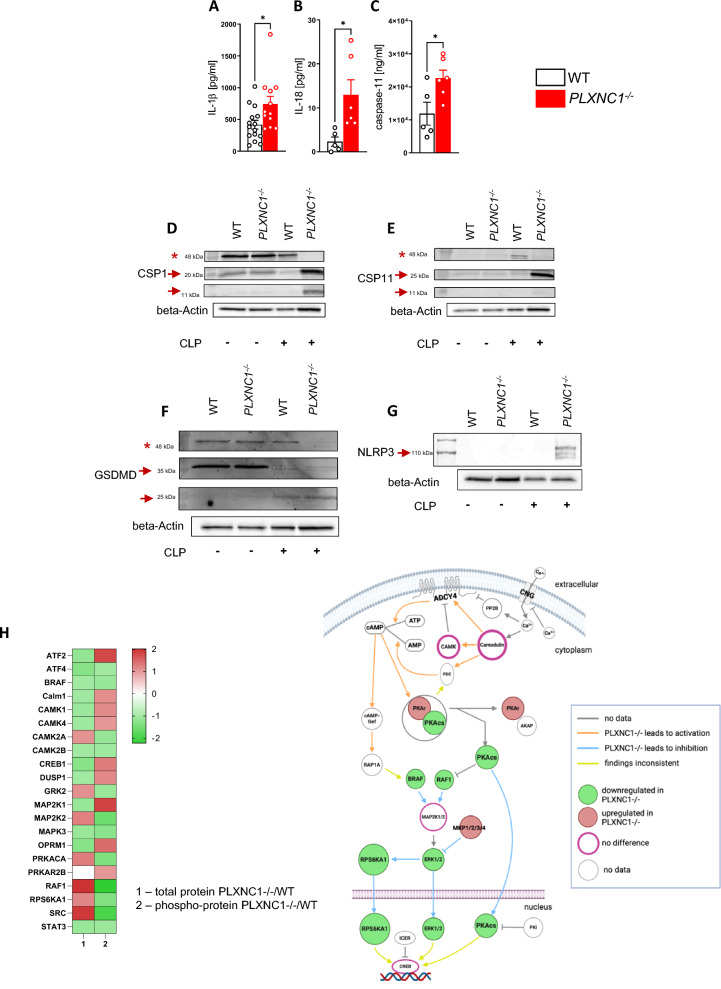
PLXNC1 alters expression of hallmark inflammasome proteins after CLP. Levels of **A** IL-1ß **B** IL-18 and **C** caspase-11 in the peritoneal lavage of *PLXNC1*^-/-^ animals and littermate controls. Western blot analysis of **D** caspase-1, **E** caspase-11, **F** gasdermin D, and **G** NLRP3 in *PLXNC1*^-/-^ animals during sepsis. (Asterisks mark the proform of caspase-1, caspase-11, and GSDMD, respectively. Arrows point at the cleaved p20 and p10 fragments of caspase-1 and caspase-11, respectively, cleaved GSDMD at around 31 kDa and 22 kDa, and full form of NLRP3.) **H** Phospho Explorer Antibody Array 24 h after CLP in *PLXNC1*^-/-^ mice and littermate controls during CLP induced sepsis. (The data are presented as the means ± SEMs; n = 5–11 mice/group; Western blots, n = 3 mice/group; *p < 0.05; **p < 0.01 and ***p < 0.001 as indicated. The illustration for Fig. 2H has been created using biorender.com.) ADCY4, Adenylate cyclase 4; AKAP, A-kinase anchoring protein; AMP, adenosine monophosphate; ATF2/4, Activating transcription factor 2/2; ATP, adenosine triophosphate; BRAF, B-rapidly accelerated fibrosarcoma; cAMP, cyclic adenosine monophosphate; cAMP-Gef, cAMP-activated guanine nucleotide exchange factor; Calm1, Camodulin-1; CAMK1/2A/2B/4, Calcium/Calmodulin-dependent protein kinase type 1/2A/2B/4; CNG, cyclic nucleotide-gated channel; DUSP1, Dual specificity phosphatase 1; ERK1/2, Extracellular signal-regulated kinase 1/2; GRK2, G-protein coupled receptor 2; ICER, Inducible cAMP early repressor; MAP2K1/2, Mitogen-activated kinase kinase 1/2; OPRM1, Opioid receptor Mu 1; PKAcs, Protein kinase A catalytic subunit; PDE, phosphodiesterase; PKAr, Protein kinase A regulatory subunit; PKI, Protein kinase inhibitor peptide; PRKACA, Protein kinase cAMP-activated catalytic subunit alpha; PRKAR2B, cAMP-dependent protein kinase type-II-beta regulatory subunit; RAF1, Rapidly accelerated fibrosarcoma-1; RAP1A, Ras-related protein 1A; RPS6KA1, Ribosomal protein S6 kinase alpha-1; SRC, Tyrosine protein kinase Src; STAT3, Signal transducers and activators of transcription

IL-1β and IL-18 are hallmark cytokines of inflammasome activation, and levels of both were significantly increased in the peritoneal lavage fluid of *PLXNC1*^-/-^ animals 24 h after CLP (Fig. 2A, B). IL-1β levels were measured in homogenized intestine and kidney and were significantly higher in intestinal (Additional file 1: Fig. S1A) and renal tissue (Additional file 1: Fig. S1B) of *PLXNC1*^-/-^ mice, indicating excessive inflammasome-mediated organ damage at these sites.

Protein levels of caspase-11—key protein for identifying intracellular LPS of gram-negative bacteria—was significantly higher in the lavage fluid of *PLXNC1*^-/-^ animals (Fig. 2C), hinting at a suppressive function of PLXNC1 in this context. In cells isolated from peritoneal lavage fluid, Western blot analysis was performed for caspase-1, caspase-11 and effector protein gasdermin D (GSDMD) (Fig. 2D–F). Inactive pro-caspase-1 appears at 45 kDa (asterisk), active p20- and p10-subunits for caspase-1 appear at 20 kDa and 10 kDa (arrows), respectively. The p20- and p10-subunit of caspase-1 is most prominent for CLP-treated *PLXNC1*^-/-^ mice (Fig. 2D). For caspase-11 (Fig. 2E), the proform appears at around 45 kDa (asterisk), and is most prominent in WT after CLP treatment, but the active p20- and p10-subunits at around 20 kDa and 10 kDa (arrows) are strongest in *PLXNC1*^-/-^ animals after CLP, suggesting a more intense cleavage of caspase-11 in the absence of PLXNC1. Effector protein gasdermin D (GSDMD) (Fig. 2F) shows its full—inactive—form at 51 kDa (asterisk), and is then cleaved into an active N-terminal fragment, which appears around 35 kDa (upper arrow), and a C-terminal fragment sized 22 kDa (lower arrow). Sham surgery (first two samples) provoked the appearance of the N-terminal fragment in both genotypes with no difference between groups. After CLP, Western blot analysis revealed the C-terminal fragment at around 22 kDa; said fragment was more intense in *PLXNC1*-/- samples. When blotting for the NLRP3 inflammasome (Fig. 2G), NLRP3 only appears at 110 kDa (arrow) in samples of *PLXNC1*-/- animals who underwent the CLP procedure; no NLRP3 can be detected in other samples.

Phosphoprotein analysis on peritoneal lavage fluid revealed a significant change in cyclic adenosine monophosphate (cAMP) signaling in *PLXNC1*^-/-^ mice: phosphorylation of SCR, a tyrosine protein kinase which is activated by protein kinase A through phosphorylation, is lower in *PLXNC1*-/- samples. As SCR is required for cAMP activation [28], this finding predicts a decreased activity of cAMP signaling in *PLXNC1*-/-. Additionally, the protein kinase A katalytic subunit (PKAcs) downstream of the cAMP signaling is predicted to be downregulated, as well as RPS6KA1, another kinase downstream of cAMP signaling, which mediates phosphorylation of transcription factor CREB [29] (Fig. 2H). Collectively, these results indicate a role for PLXNC1 in the regulation of the noncanonical inflammasome in our in vivo model.

### *Plexin C1 alters the immune response to intracellular LPS *in vivo* and *ex vivo

**Fig. 3:**
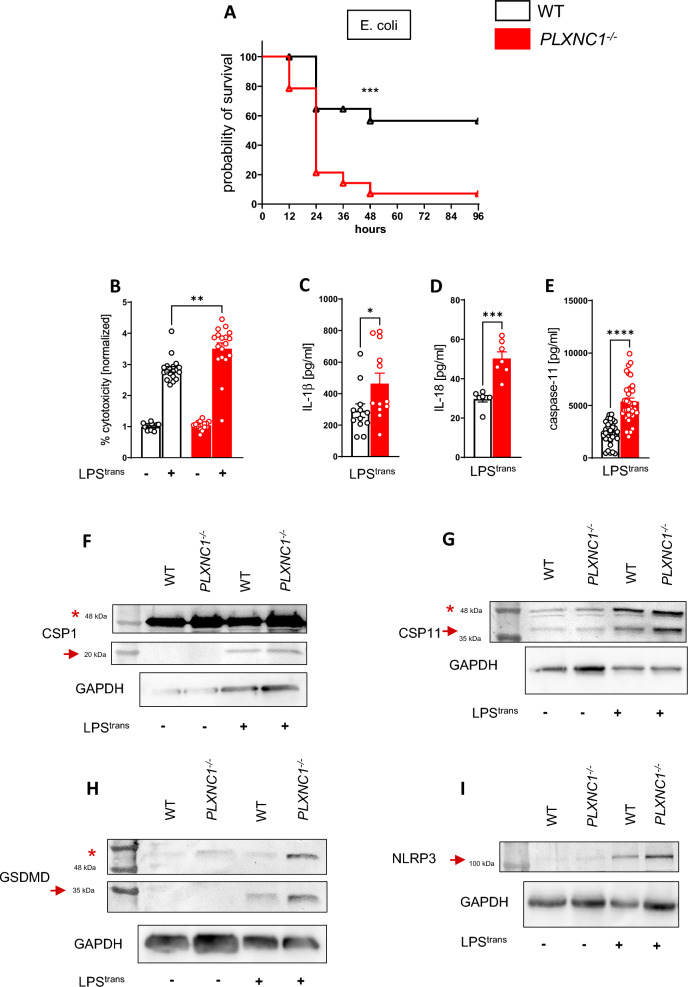
PLXNC1 is involved in intracellular LPS processing in vivo and ex vivo. *PLXNC1*^-/-^ mice and littermate controls were exposed to intraperitoneal E. coli injection and survival was monitored. **A** *PLXNC1*^-/-^ animals and littermate controls survival curves after intraperitoneal injection of viable *E. coli*. BMDMs were isolated from *PLXNC1*^-/-^ mice and their littermate controls and transfected with LPS to activate caspase-11. The levels of **B** LDH, **C** IL-1ß, **D** IL-18 and **E** caspase-11 were measured in cell culture supernatant through ELISA. In lysed cells protein of respective LPS transfected BMDMs, **F** caspase-1 (asterisk marks the full form at around 50 kDa; arrow points at cleaved caspase-1 p20), **G** caspase-11 (asterisk marks 43 kDa; arrow points at 38 kDa), **H** gasdermin D (asterisk marks full form at around 50 kDa; arrow points at cleaved gasdermin D at around 31 kDa) and **I** NLRP3 expression (arrow points at full size receptor at around 110 kDa) was examined through Western blot analysis. (The data are presented as the means ± SEMs; n = 5–18 mice/group; Western blots, n = 4–7 mice/group; *p < 0.05; **p < 0.01 and ***p < 0.001 as indicated)

To further examine the role of PLXNC1 in vivo, we injected live E. coli bacteria, a common model to specifically induce gram-negative sepsis [30]. Outer membrane vesicles (OMV) of bacteria ensure intracellular uptake of LPS and caspase-11 activation [31, 32]. Probability of survival was significantly decreased for *PLXNC1*^-/-^ mice here (Fig. 3A). When mice received an i.p. injection of pure LPS (from *E. coli* subtype O111:B4), which is mainly recognized by the membrane receptor TLR4 [33], however, we found no difference in survival between PLXNC1^-/-^ animals and littermate controls (Additional file 1: Fig. S2A).

For specific molecular workup, we isolated BMDMs from *PLXNC1*^-/-^ mice and littermate controls and activated caspase-11 ex vivo by LPS transfection (from *E. coli* subtype O111:B4). In the supernatant of *PLXNC1*-/- samples, levels of LDH to quantify percentage of cell death, IL-1β, IL-18, and caspase-11 were significantly higher (Fig. 3B–E). (Baseline levels of IL-1β, IL-18, and caspase-11 for unstimulated controls were below detection threshold.)

In addition, Western blot analysis of cell lysates for caspase-1, caspase-11, GSDMD, and NLRP3 was carried out (Fig. 3F–I; Additional file 1: Fig. S3A–D).

For caspase-1, procaspase-1 was strongest in *PLXNC1*-/- after LPS transfection (Fig. 3F, asterisk; Additional file 1: Fig. S3A), as was the active p20-subunit at 20 kDa (Fig. 3F, arrow). For caspase-11 expression (Fig. 3G; Additional file 1: Fig. S3B), a larger fragment appears at around 43 kDa (asterisk) and another fragment at 38 kDa (arrow); the 38 kDa fragment was most prominent in *PLXNC1*-/- samples after LPS transfection (Fig. 3G; Additional file 1: Fig. S3B). For GSDMD (Fig. 3H; Additional file 1: Fig. S3C), the full form appears at around 50 kDa (Fig. 3H, asterisk), and the active N-terminal fragment at around 31 kDa (Fig. 3H, arrow). In accordance with our previous results, the active N-terminal fragment is strongest in *PLXNC1*-/- samples after LPS transfection (Additional file 1: Fig. S3C), pointing at a more severe expression of the noncanonical inflammasome in the absence of PLXNC1.

As the NLRP3 inflammasome is known to be activated upon LPS transfection in a noncanonical manner, we also blotted for the NLRP3 receptor, and found a higher expression in *PLXNC1*-/- samples as a reaction to LPS transfection when compared to wild-type samples (Fig. 3I, arrow; Additional file 1: Fig. S3D).

This finding indicates an inhibitory effect of PLXNC1 on the caspase-11-mediated response to LPS. When using lipid A—the core component of LPS—we achieved comparable results: levels of LDH and IL-1β (Additional file 1: Fig. S2A-B) in the cell culture supernatants of *PLXNC1*-/- BMDMs were increased. Additionally, we stimulated BMDMs with nigericin and flagellin, for independent activation of the NLRP3, and NLRC4 inflammasome, respectively, but found no difference between genotypes when we measured LDH and IL-1β levels in cell culture supernatant (Additional file 1: Fig. S2D-G).

### PLXNC1 controls the immune response to intracellular LPS by interfering with the protein kinase A signaling pathway

**Fig. 4:**
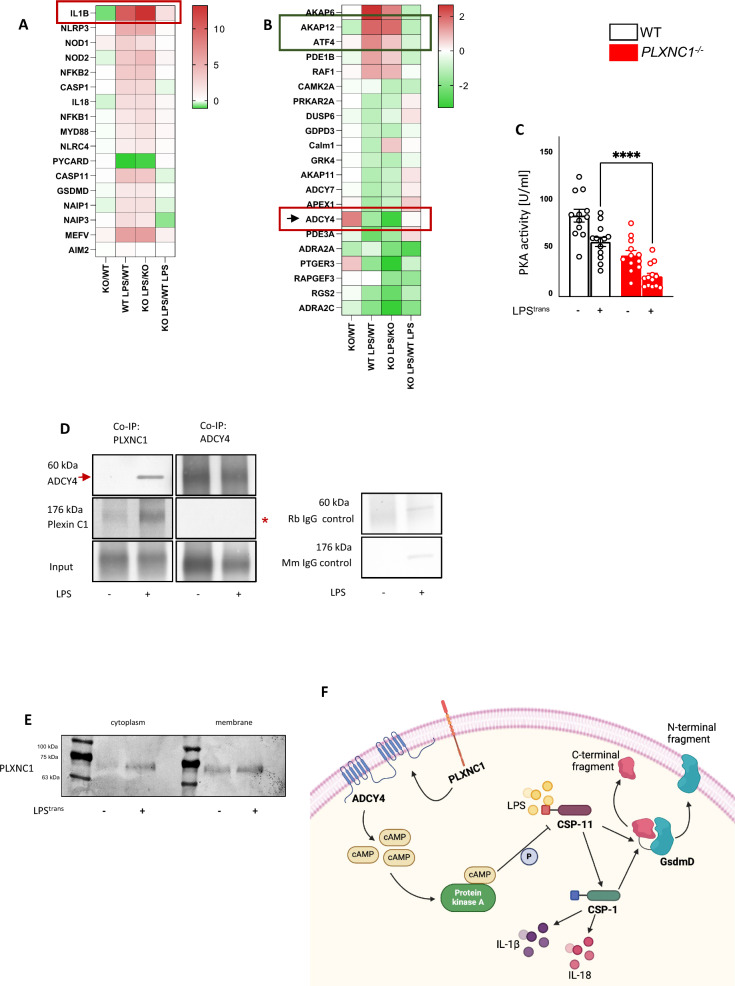
PLXNC1 alters immune response to intracellular LPS through a mechanism based on cAMP metabolism and interaction with adenylate cyclase 4 (ADCY4). Next-generation sequencing (NGS) was performed on BMDMs from *PLXNC1*^-/-^ mice and their littermate controls after LPS transfection. Analysis of **A** the inflammasome pathway on the mRNA expression levels of inflammasome-related genes. **B** The cAMP-mediated signaling pathway in BMDMs from *PLXNC1*^-/-^ mice; notably, the mRNA expression of ADCY4 (red frame) in *PLXNC1*-/- cells under baseline conditions, and upon LPS differs. **C** PKA activity upon LPS transfection in BMDMs from *PLXNC1*-/- and wild type BMDMs. **D** Human monocytes/macrophages were transfected with LPS, and Co-immunoprecipitation was performed: samples were precipitated for PLXNC1, and blotted for ADCY4, as well as precipitated for PLXNC1 and blotted for ADCY4 (Input: a sample of the total protein lysate taken before the immunoprecipitation step; respective molecular weight of the proteins have been added to the left of the blot). **E** Digitonin assay with wild-type BMDMs revealed the presence of intracellular PLXNC1, which was enriched upon LPS transfection. **F** PLXNC1 interacts with ADCY4 to inhibit caspase-11 activation through a mechanism based on cAMP-metabolism and PKA activity (The data are presented as the means ± SEMs; n = 4–11 mice/group; Western blots, n = 3 mice/group; *p < 0.05; **p < 0.01 and ***p < 0.001 as indicated). The illustration for Fig. 4F was created using biorender.com. ADCY4, Adenylate cyclase 4; cAMP, cyclic adenosine monophosphate; CSP1/11, Caspase-1/-11; GsdmD, Gasdermin D; IL-1β, Interleukin-1β/-18; LPS, lipopolysaccharide; mM, mus musculus; Rb, rabbit

To gain further insight into the mechanisms by which PLXNC1 controls the response to cytoplasmatic LPS, we transfected BMDMs with LPS and performed RNA sequencing analysis to identify altered cell signaling pathways. Surprisingly, except for IL-1β, the transcription of genes controlling the noncanonical inflammasome pathway was not upregulated in *PLXNC1*-/- BMDMs when compared to littermate controls (Fig. 4A; Additional file 1: Fig. S4A). Therefore, we investigated pathways known to control caspase-11 activity.

Among those pathways, activation of protein kinase A (PKA) has been shown to restrain caspase-11 activity in a manner which depends on the ADRA2B-ADCY4-PDE8A-PKA axis. Under physiological conditions, levels of cAMP and PKA decrease after stimulation of caspase-11 by intracellular LPS; a rest activity remains, however, and restrains caspase-11 activity [5]. Inactivation of PKA therefore results in excessive caspase-11 activity [10].

NGS results of the PKA signaling pathway indeed revealed interesting differences between genotypes: Notably, expression of adenylate cyclase 4 (ADCY4) is altered between *PLXNC1*-/- and wild type samples (Fig. 4B, red frame). ADCY4 increases the intracellular level of cAMP, which in turn activates PKA [10]. Our NGS results show that under baseline conditions [KO/WT] (Fig. 4B, red frame, first box), ADCY4 gene transcription is higher in *PLXNC1*-/- when compared to littermate controls. Furthermore, in our wild type cells, ADCY4 is accordingly downregulated upon LPS transfection [WT LPS/WT], but due to the different baseline levels, the downregulation of ADCY4 is more intense in *PLXNC1*-/- cells [KO LPS/KO], thus indicating a greater decline in PKA activity than normally. There is no difference between genotypes for the direct comparison ‘KO LPS/WT LPS’, however, leaving us to speculate that the higher caspase-11 expression in *PLXNC1*-/- is caused by the difference between baseline expression and expression after LPS transfection (Fig. 4B, red frame; Additional file 1: Fig. S4B, red frame).

A-kinase anchor protein 12 (AKAP12) is a scaffolding complex interacting with and regulating the activity of PKA, causing a variety of effects [34, 35]. Our data show that AKAP12 transcription is slightly lower *in PLXNC1*-/- samples compared to WT samples under baseline conditions (Fig. 4B, green frame, KO/WT); transcription is upregulated upon LPS transfection in both WT and PLXNC1-/- samples, but altogether lower in *PLXNC1*-/- cells (Fig. 4B, green frame, KO LPS/WT LPS). This points at an altered—and possibly diminished—activity of PKA in *PLXNC1*-/- cells upon LPS transfection. Additionally, ATF4—a protein *downstream* of PKA-signalling, which is phosphorylated by PKA [36, 37]—shows similar changes in our experimental context: after LPS transfection, upregulation of its transcription is not as high in *PLXNC1*-/- as it is in WT samples (Fig. 4B, green frame; KO LPS/WT LPS).

Other pathways regulating caspase-11 activity—complement signaling (Additional file 1: Fig. S5A), interferon signaling (Additional file 1: Fig. S5B), and Toll-like receptor signaling (Additional file 1: Fig. S6)—didn't differ between genotypes.

### PLXNC1 directly interacts with ADCY4

To evaluate whether the difference in ADCY4-downregulation holds functional importance, we investigated whether LPS-induced reduction of PKA activity differs between WT and *PLXNC1*-/- cells. BMDMs were transfected with LPS, and PKA activity levels were measured. Figure 4C shows the difference in PKA activity between WT and *PLXNC1*-/-: Baseline activity of PKA was higher in WT; in both genotypes, LPS transfection led to significant decrease of PKA activity, but the activity was altogether significantly lower in *PLXNC1*-/-.

To evaluate the interaction between PLXNC1 and ADCY4, we performed coimmunoprecipitation (Co-IP) (Fig. 4D) of PLXNC1 and ADCY4. When precipitating PLXNC1, and blotting for ADCY4, the respective bands appeared (arrow). When precipitating ADCY4 and blotting for PLXNC1 (asterisk), however, no band appeared, which could be caused by lower protein expression of PLXNC1.

Our data, overall, shows a binding of the two proteins, providing further evidence that regulation of caspase-11 expression via PLXNC1 is directly mediated through ADCY4. PLXNC1 is a membrane-bound receptor, but using a digitonin assay on BMDMs of littermate control mice, we showed that PLXNC1 can also be present in the cytoplasm (Fig. 4E). Additionally, when treating cells with an ADCY4-blocking peptide during LPS transfection, cell death—as quantified by LDH—as well as levels of IL-1β, and IL-18 no longer differ between *PLXNC1*-/- and wild type samples (Additional file 1: Fig. S7A-C).

Overall, ADCY4 might be restrained by PLXNC1 under baseline condition and could result in a moderate decrease in response to LPS transfection, to dampen caspase-11 activity through PKA. In the absence of PLXNC1, the decrease of PKA after LPS stimulation is more intense Fig. 4F shows a simplified schematic of the mechanism by which PLXNC1 might alter immune response to intracellular LPS.

### The Plexin C1-binding protein SL4c-d suppresses immune response to intracellular LPS and improves sepsis survival

Sema-loop-4c-d (SL4c-d) is the peptide core of Semaphorin 7A, an activating ligand of PLXNC1 [21]. Investigating whether SL4c-d could therefore help reduce the sequelae of sepsis, we pretreated wild-type murine BMDMs with SL4c-d before LPS transfection. To ensure intracellular protein uptake, we transfected control samples with β-galactosidase and stained respective samples for β-galactosidase afterwards (Additional file 1: Fig. S8A).

**Fig. 5:**
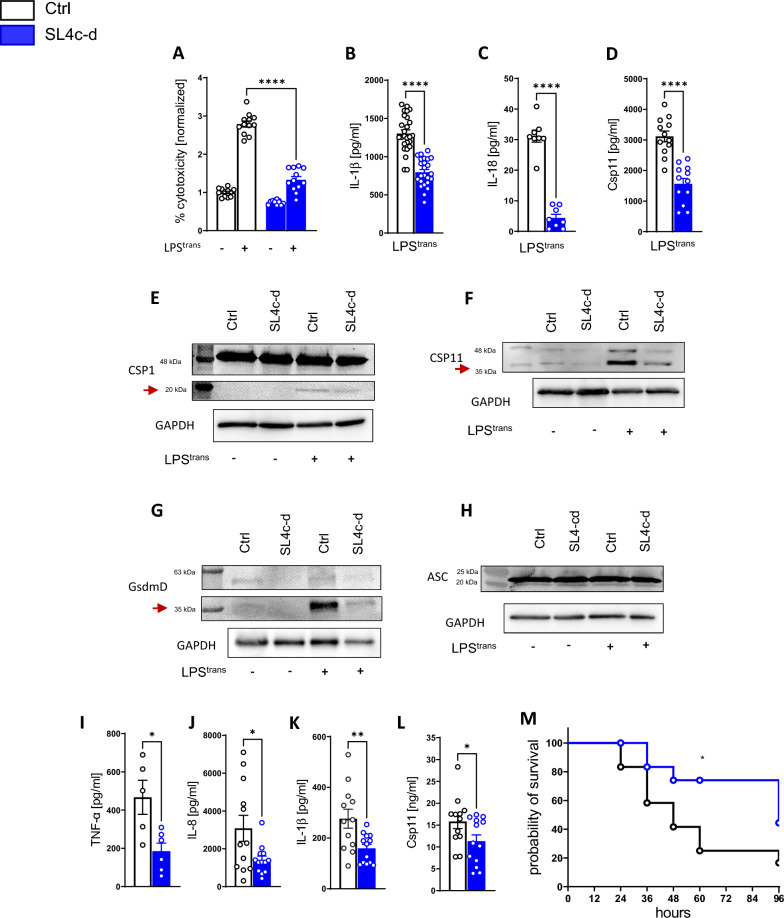
The PLXNC1 agonist SL4c-d suppresses caspase-11 activity and improves survival in polymicrobial sepsis. BMDMs from wild-type mice were transfected with the Sema-loop 4c-d peptide (SL4c-d)—a *PLXNC1* ligand—prior to LPS priming and transfection to control caspase-11 activity. In the supernatant of cells pretreated with SL4c-d, **A** LDH release and the **B** levels of IL-1β, **C** IL-18, and **D** caspase-11 and of vehicle controls. In cell lysates, the expression of **E** caspase-1 (arrow points at cleaved caspase-1 p20), **F** caspase-11 (arrow points at 38 kDa), and **G** gasdermin D protein (arrow points at cleaved gasdermin D at 31 kDa) was decreased after SL4c-d treatment, while **H** ASC protein expression was unaffected. Polymicrobial midgrade sepsis was induced in mice by CLP, and the mice received a daily i.v. injection of SL4c-d bound to Arg9 to ensure intracellular peptide delivery. After 24 h, the levels of the cytokines **I** TNF-α and **J** IL-8 were measured in the peritoneal lavage fluid from SL4c-d-treated animals. The levels of **K** IL-1β and **L** caspase-11 in the peritoneal lavage fluid were also measured. **M** Survival time of mice receiving SL4c-d treatment or vehicle control during polymicrobial sepsis over the course of 96 h. (The data are presented as the means ± SEMs; n = 4–11 mice/group; Western blots, n = 3 mice/group; *p < 0.05; **p < 0.01 and ***p < 0.001 as indicated)

Pretreatment with SL4c-d led to significantly decreased levels of LDH to quantify the percentage of cell death, IL-1β, IL-18, and caspase-11 (Fig. 5A–D) in cell culture supernatant, indicating a decreased activity of the noncanonical inflammasome.

Western blot analysis of cell lysates also showed lower expression of the p20-fragment of caspase-1 (Fig. 5E, arrow; Additional file 1: Fig. S9A) after SL4c-d treatment. Caspase-11appears at around 43 kDa and 38 kDa (Fig. 5F; Additional file 1: Fig. S9B), and upon LPS transfection, SL4c-d treated samples show lower expression especially of the 38 kDa fragment. Accordingly, the N-terminal fragment of active GSDMD at around 31 kDa was lower in SL4c-d-treated samples (Fig. 5G; Additional file 1: Fig. S9C). Taken together, these findings indicate that SL4c-d dampens inflammasome expression upon recognition of intracellular LPS.

Expression of the adaptor molecule ASC remained unchanged (Fig. 5H; Additional file 1: Fig. S9D). We also detected no difference in Western blot analysis of NLRP3 (Additional file 1: Fig. S7B).

BMDMs of *PLXNC1*^-/-^ mice showed no significant differences in LDH release as a sign of cytotoxicity (Additional file 1: Fig. S8C) or IL-1β (Additional file 1: Fig. S8D) after LPS transfection when pre-treated with SL4c-d compared to control samples.

For in vivo experiments, we again employed the CLP model. SL4c-d was bound to Arg9 to ensure intracellular uptake (Additional file 1: Fig. S10). Wild-type mice were subjected to CLP and received an i.v. injection of SL4c-d. After 24 h, levels of IL-1β, TNF-α, IL-8, and caspase-11 were significantly lower (Fig. 5I-L) in the group which received SL4c-d. During survival experiments, mice in the treatment group received an i.v. injection of SL4c-d as described above every 24 h; mice in the placebo group received a control injection at the same time points. SL4c-d-treated animals had a significant survival advantage (Fig. 5N). Taken together, our in vivo findings suggest that SL4c-d is a potential therapeutic tool for sepsis and might help suppress inflammatory damage through a PLXNC1-dependent pathway (Additional file 2).

## Discussion

Inflammatory response to intracellular LPS as it occurs during infection with gram-negative bacteria can cause detrimental organ damage. Therefore, understanding the underlying molecular mechanisms might hold the key to improvement in treating gram-negative sepsis.

It is known that the inflammasome as an innate immune response is part of the front-line defense. In short, the inflammasome consists of different pattern recognition receptors, which—after binding their respective ligands—open into a common final path leading to activation of caspase-1 and further on, to excretion of proinflammatory cytokines IL-1β, and IL-18 as well as pore-formation in the cell membrane mediated by gasdermin D. This pore-formation causes a lytic, highly inflammatory form of cell death termed pyroptosis [38]. Caspase-11 is the plasmatic sensor for LPS, and after activation via LPS-binding, caspase-11 is able to cleave GSDMD and thus initiate pyroptosis independently of caspase-1 [6, 39]. Through cross-talk with NLRP3, caspase-11 is also capable of caspase-1 activation—which is necessary for cleavage of pro-IL-1β and pro-IL-18 [6]. We aimed to identify possible mechanisms to control the immune response to intracellular LPS and to eventually improve treatment of LPS-based infection, and found the neuronal guidance receptor PLXNC1 to be a valuable target.

In our in vivo model of polymicrobial sepsis, the absence of PLXNC1 resulted in a more severe inflammatory immune response, markedly with higher levels of IL-1β and IL-18, the hallmark cytokines of inflammasome activity. Additionally, caspase-11 expression was upregulated, as was caspase-1 and gasdermin D. A similar response could be reproduced in cell culture experiments with murine BMDMs after LPS transfection: We found IL-1β and IL-18 to be significantly higher in samples lacking PLXNC1, and in accordance, protein expression of cleaved caspase-1, activated gasdermin-D, NLRP3 and higher levels of caspase-11, altogether indicating an unrestrained immune response to intracellular LPS.

So far, inflammasome response to LPS and consecutive treatment possibilities are not understood well enough to be of clinical relevance, though respective effort has been made. Previous research has discovered that caspase-11 is indirectly influenced by glutathione peroxidase-4 (GPX-4), which negatively regulates the caspase-11 substrate gasdermin D, and a lack of GPX-4 resulted in increased organ injury [40]. Oxidized PAPC (1-palmitoyl-2-arachidonoyl-sn-glycero-3-phosphocholine) binds to caspase-11 and activates the IL-1 response, whereas pyroptosis is not promoted [41]. Stearoyl lysophosphatidylcholine, a component of oxidized lipoproteins, inhibits binding of LPS to caspase-11 and diminishes the cellular response to cytosolic LPS [42].

A control mechanism with a direct suppressive effect on caspase-11 activity is the metabolism of cAMP. During binding of caspase-11 to LPS, ADCY4-mediated cAMP production leads to altered PKA activity, and PKA subsequently inhibits caspase-11 activity, resulting in decreased IL-1ß and IL-18 levels and reduced cytotoxicity [10]. Influencing this response mechanisms, PLXNC1 is exerting its effect. We are the first to report that a receptor belonging to the class of neuronal guidance proteins (NGPs)—PLXNC1—controls the immune response to intracellular LPS through PKA-signaling. It appears that PLXNC1 interacts with ADCY4 under homeostatic conditions already, containing its expression, so that when ADCY4 and PKA, subsequently, are downregulated upon intracellular LPS stimulation, decrease in activity stays within a certain level to restrain caspase-11 response. Treatment with the PLXNC1 ligand SL4c-d dampened the immune response to LPS transfection and CLP, with significantly decreased levels of IL-1β and IL-18 and improved CLP-survival.

An immune-modulating effect of NGPs was described, yet no work exists to date that has shown a role for plexins in sepsis or sepsis-related inflammasome regulation. This finding is novel and shows that a previously unknown class of proteins is involved in the control of the inflammasome response. Previously, a role for PLXNC1 during the migration of immunocompetent cells such as neutrophils has been reported [18]. The capacity of PLXNC1 to serve as a stop signal is also shown in macrophages during the development of pulmonary fibrosis. *PLXNC1*-/- macrophages demonstrate an increased collagen accumulation and TGF-β 1 expression in the lungs following bleomycin stimulation. A deficiency of PLXNC1 enhanced the development of pulmonary fibrosis and could be ameliorated when PLXNC1 was reconstituted [22]. A role for PLXNC1 in the control of intracellular LPS sensing and its effects was, however, not described before. We demonstrate how PLXNC1 significantly regulates the immune response to intracellular LPS and might be a target for the suppression of sepsis-associated secondary organ injury in the future. Control of caspase-11 (caspase 4/-5 in humans) is proven to be a valuable target to interfere with sepsis induced damage, and we show that PLXNC1 holds the potential to control this activity. Future work should focus on a possible role of the described axis, using PLXNC1 to reduce the detrimental effects of hyperinflammation during sepsis.

## Conclusion

Based on the results we have obtained, we were able to show that the plexin C1 receptor restrains the immune response to intracellular LPS both ex vivo and in vivo. Furthermore, we demonstrate here that the activation of plexin C1 by ligand-binding during murine polymicrobial sepsis ameliorates inflammatory response and improves survival. These results show a previously unknown mechanism for PLXNC1 suppressing excessive noncanonical inflammasome activity and offer a new potential target for treatment of sepsis with its detrimental effects.

### Supplementary Information

**Additional file 1**.

**Additional file 2**.

## Data Availability

For original data, please contact peter.rosenberger@medizin.uni-tuebingen.de.

## Abbreviations

ABP: ADCY4 binding peptide
ADCY4: Adenylate cyclase 4
AST: Aspartate aminotransferase
BMDMs: Bone marrow-derived macrophages
cAMP: Cyclic adenosine monophosphate
CARD: Caspase-recruitment domain
CLP: Cecal ligation and puncture
co-IP: Coimmuniprecipitation
CREB: Cyclic AMP response binding protein
FDR: False discovery rate
GPX-4: Glutathione peroxidase-4
GSDMD: Gasdermin D
i.e.: Id est
LPS: Lipopolysaccharides
LDH: Lactate dehydrogenase
NGP: Neuronal guidance protein
NGS: Next-generation sequencing
OMV: Outer membrane vesicles
PKA: Protein kinase A
PLXNC1: Plexin C1
SL4c-d: Sema-loop-4c-d
TPM: Transcripts per million
