# Supplementary material for: Plexin C1 influences immune response to intracellular LPS and survival in murine sepsis
Source: J Biomed Sci. 2024 Aug 21;31:82. doi: 10.1186/s12929-024-01074-x (PMC11337750; doi:10.1186/s12929-024-01074-x)
Supplement: Supplementary file 1 — Additional file 1. [file 12929_2024_1074_MOESM1_ESM.pdf]

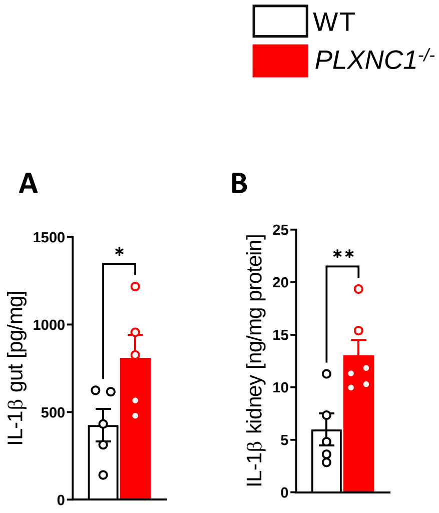

Supplemental Figure 1. IL-1β levels in lysed A) intestinal and (B) kidney tissue from animals undergoing 24 hours of CLP treatment were significantly higher in Plexin C1-deprived samples (n=6 for each analysis).

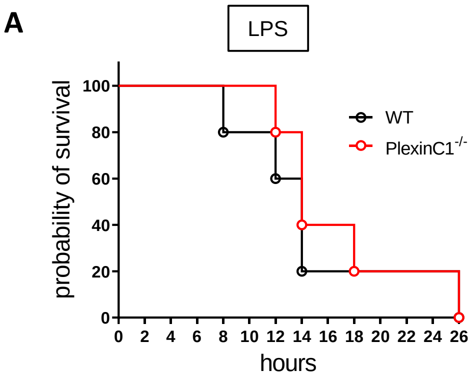

Supplemental Figure 2A . PLXNC1-/- mice and littermate controls received an i.p. injection of LPS and were monitored for survival. **(A)** Survival rates did not differ between genotypes [n = 5].

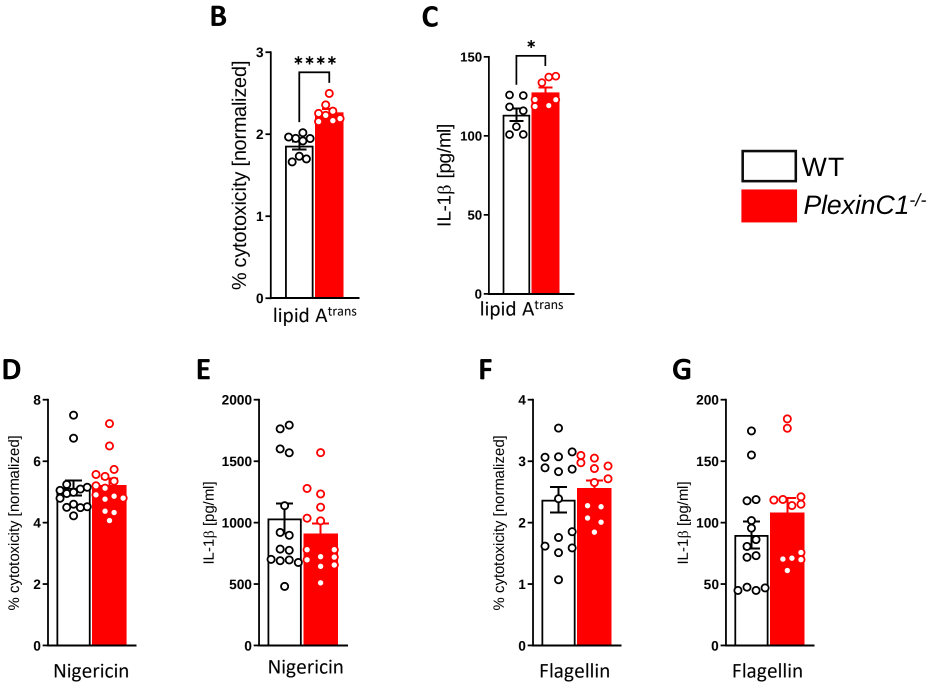

Supplemental Figure 2 B-G. Murine BMDMs were transfected with LPS core protein lipid A for caspase-11 activation, nigericin for activation of NLRP3, and flagellin for activation of NLRC4, respectively: Plexin C1-deprived samples show (B) significantly higher cytotoxicity [n=8], and (C) IL-1β levels [n=7] in cell culture supernatant. For nigericin, there was no significant difference in (D) cytotoxicity [n = 14], or (E) IL-1β [n = 14] in cell culture supernatant; for flagellin, there was no significant difference in (F) cytotoxicity [n = 14], or (G) IL-1β [n = 14] in cell culture supernatant.

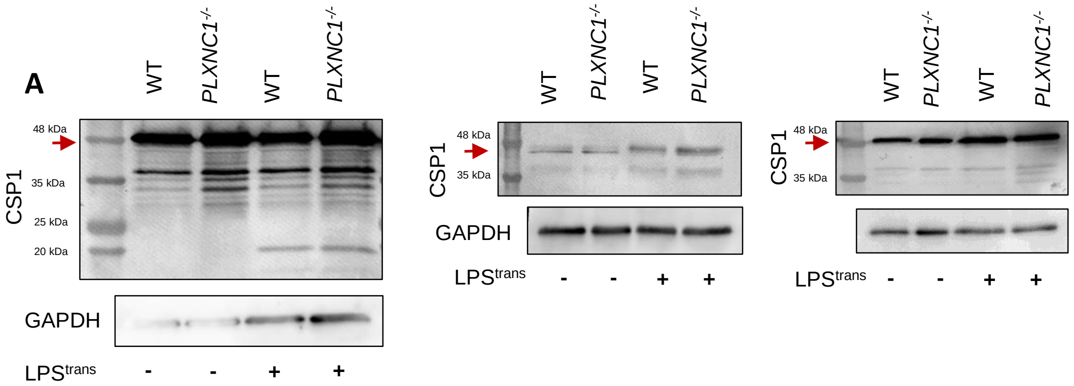

Supplemental Figure 3: (A) Western blot analysis of caspase-1 (corresponding to Fig. 3F) for three independent experiments, and

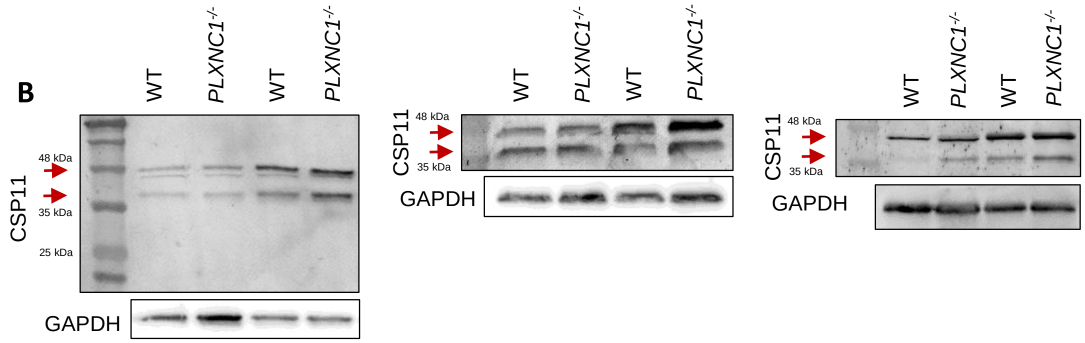

Suppl. Figure 3: (B) Western b**l**ot analysis of caspase-11 (corresponding to Fig. 3G) for three independent experiments, protein appearing at around 45 kDa and (E) 38 kDa as indicated by the antibody manufacturer.

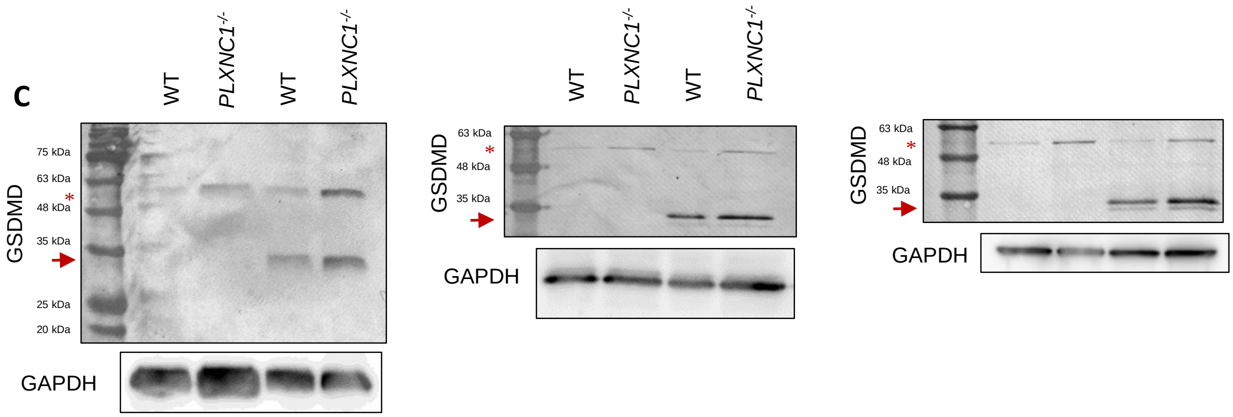

Suppl. Figure 3: (C) Western blot analysis of GSDMD (corresponding to Fig. 3E) for three independent experiments, the full sized form at around 50 kDa (asteriks), the cleaved N-terminal fragment at around 30 kDa (arrow).

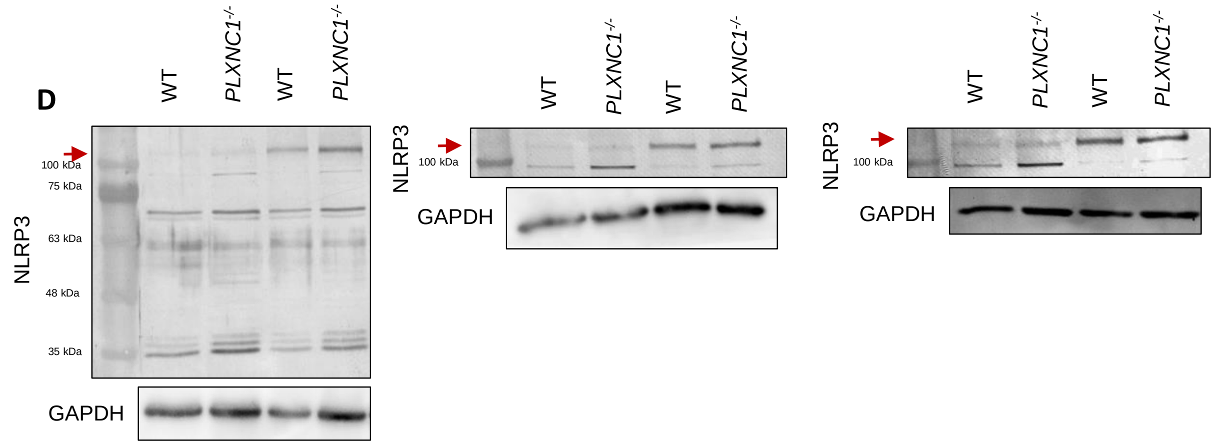

Supplemental Figure 3: (D) Western blot analysis of NLRP3 (corresponding to Fig. 3F) for three independent experiment at around 110 kDa (arrow).

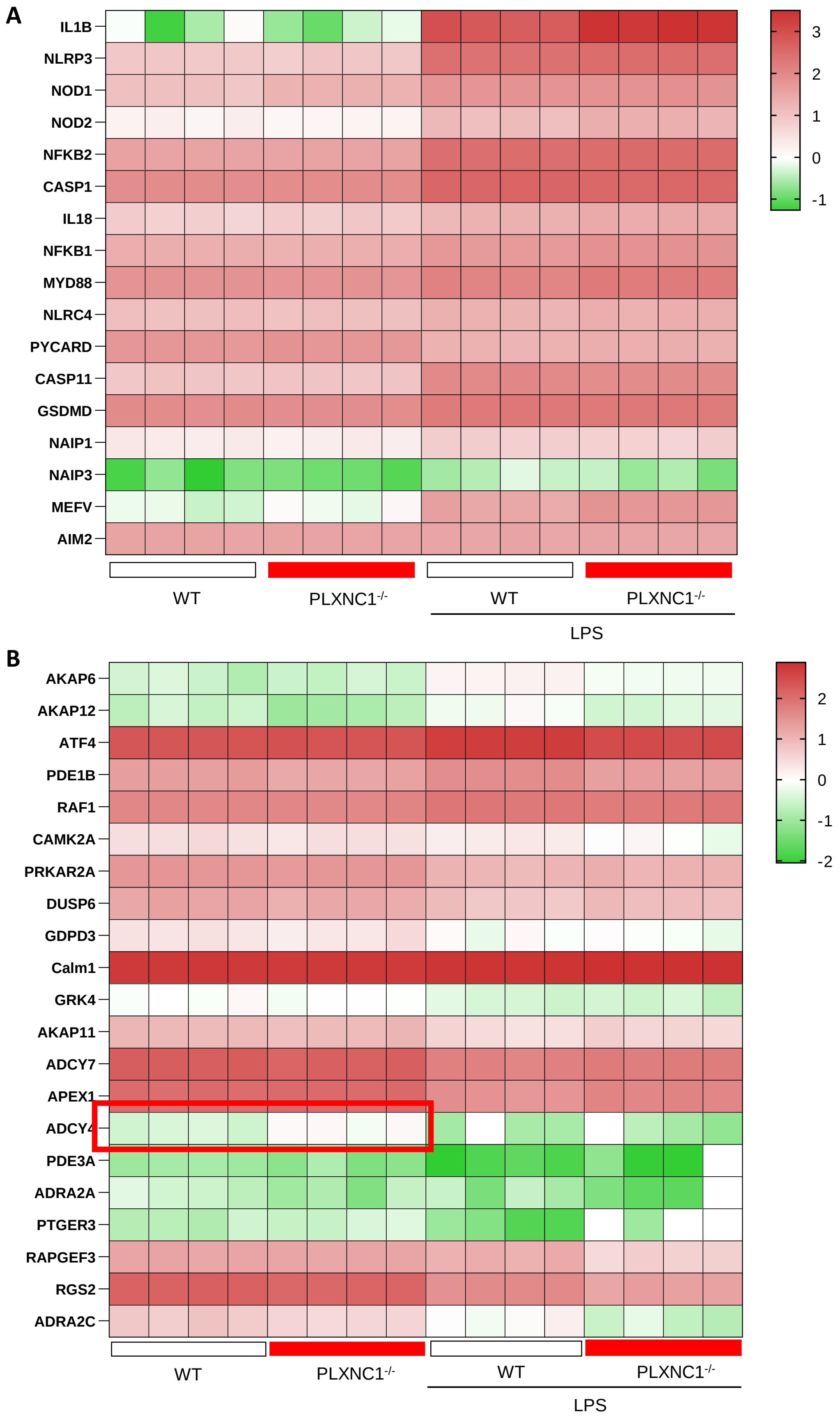

Supplemental Figure 4. Next generation sequencing of genes from (A) the inflammasome pathway and (B) cAMP signaling (n=4). Log_{10} of transcripts per kilobase million (TPM) was used.

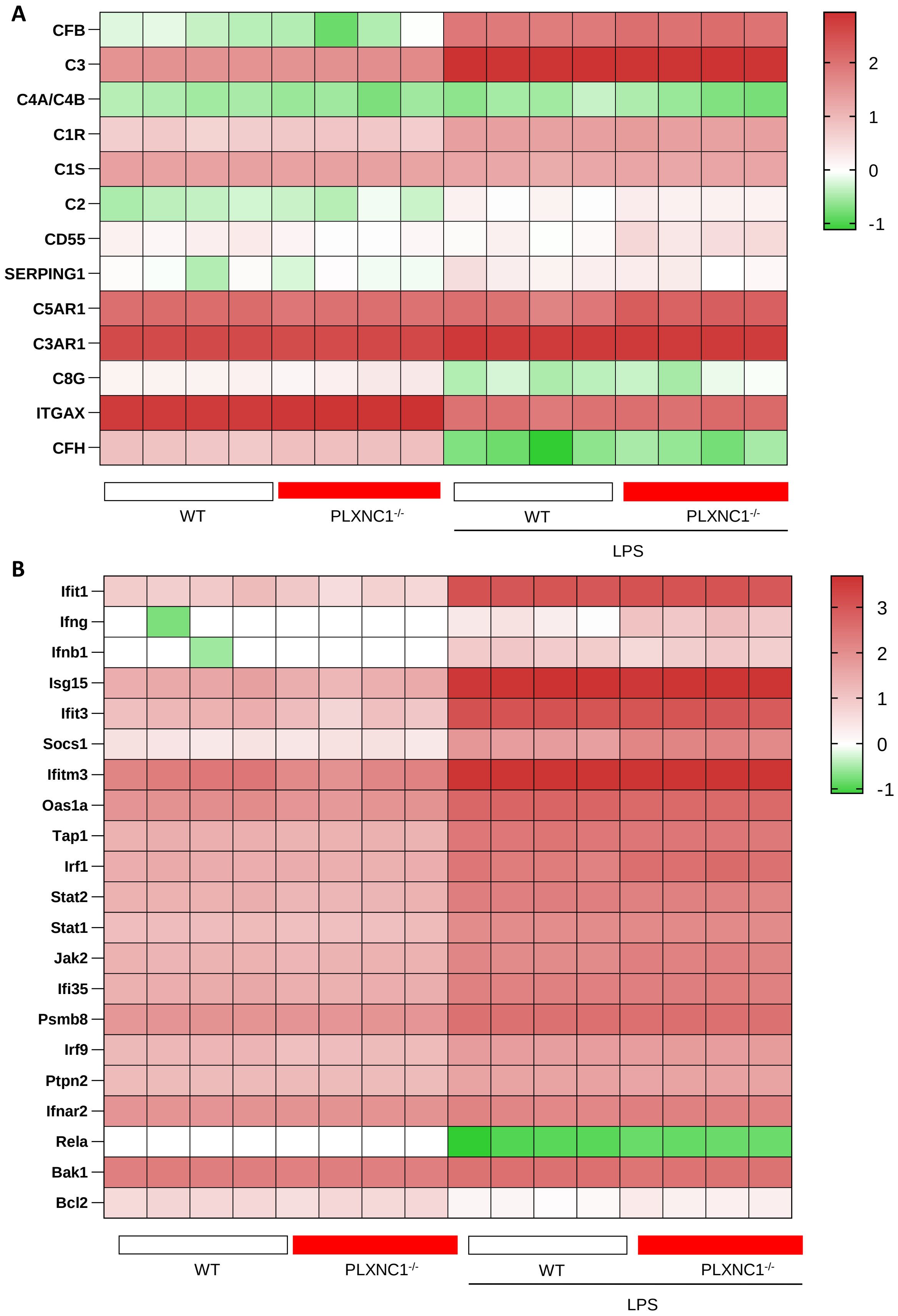

Supplemental Figure 5. Next generation sequencing of genes from (A) the complement pathway and (B) interferon signaling (n=4). Log_{10} of transcripts per kilobase million (TPM) was used.

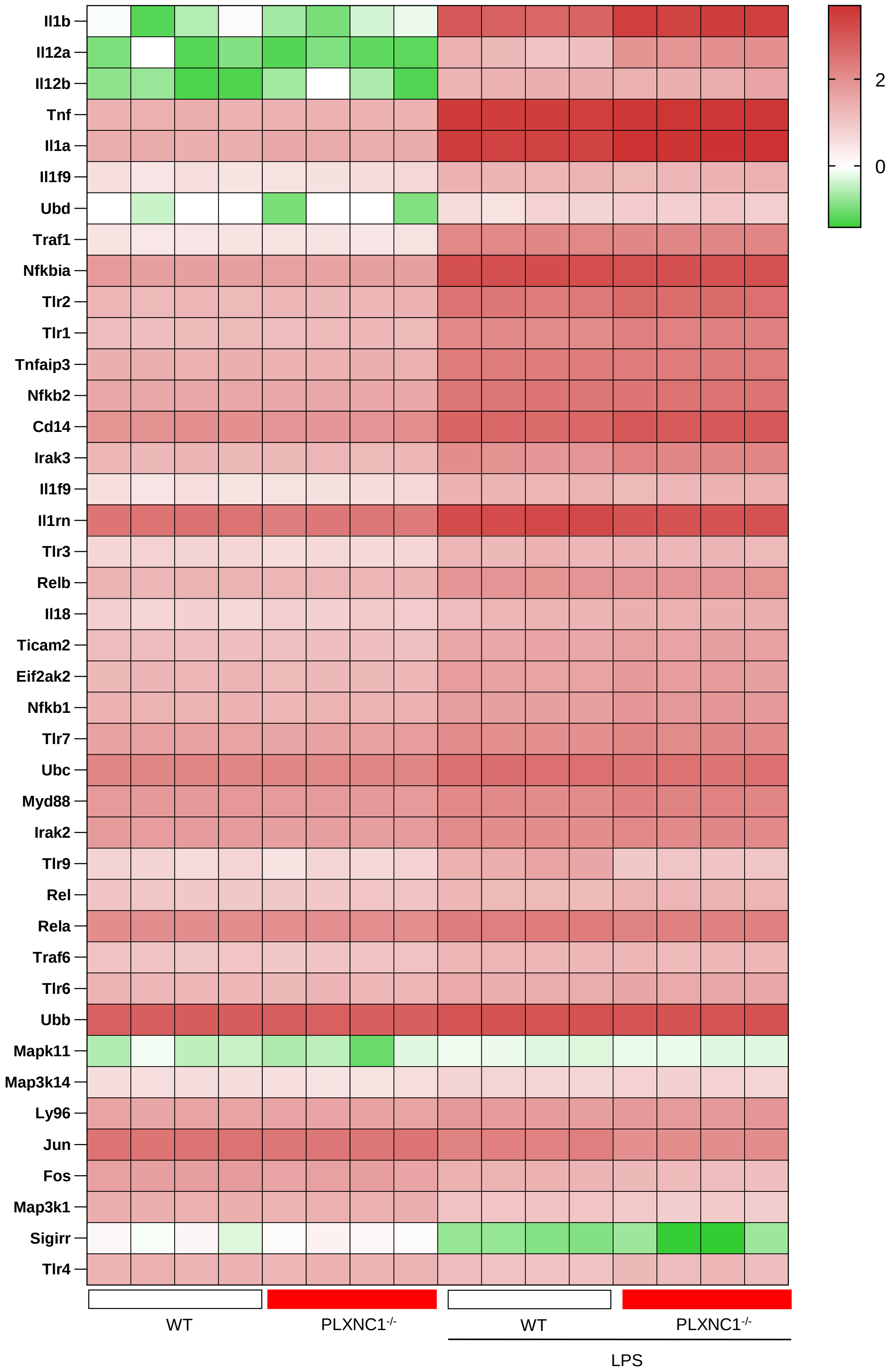

Supplemental Figure 6. Next generation sequencing of genes from (A) the complement pathway and (B) interferon signaling (n=4). Log_{10} of transcripts per kilobase million (TPM) was used.

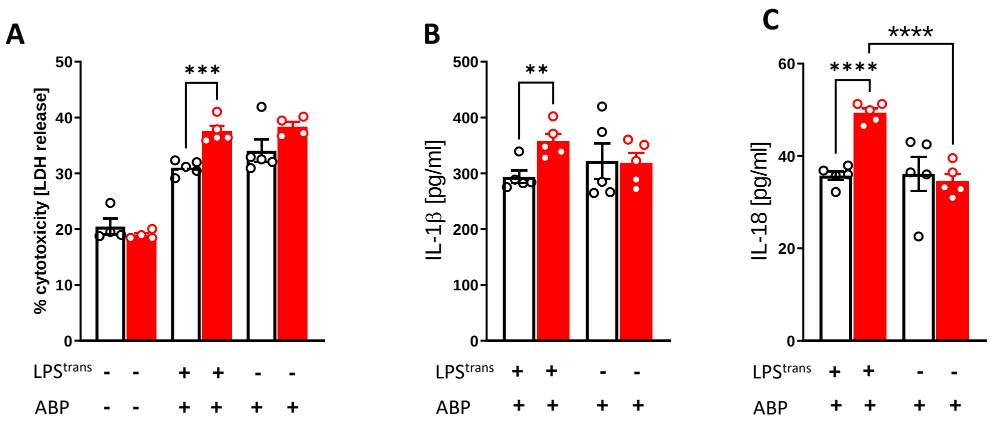

Supplemental Figure 7. Murine BMDMs of PLXNC1-/- mice and littermate controls were transfected with LPS, and ADCY4 was blocked using an ADCY4 blocking peptide (ABP). When ADCY4 was blocked, levels of (A) LDH as a quantification for cell death, (B) IL-1β, and (C) IL-18 were no longer significant between PLXNC1-/- samples and those of littermate controls after LPS transfection.

A

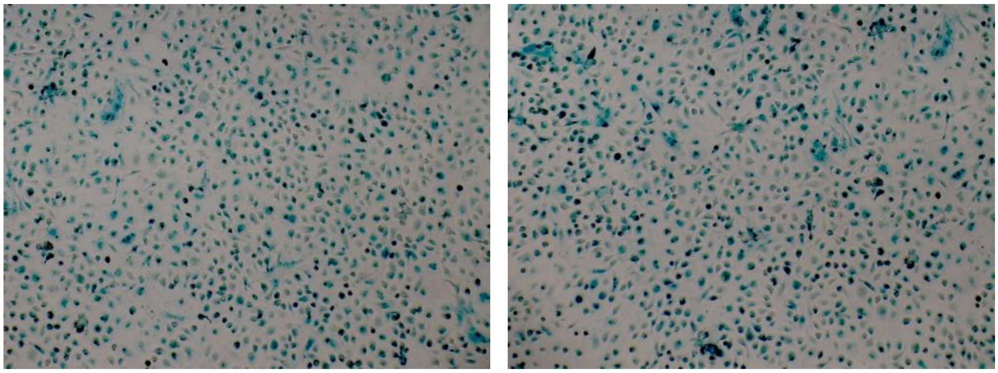

Supplemental Figure 8 (A). BDMDs of wild type mice were transfected with β-galactosidase to visualize protein uptake as a control for successful SL4c-d transfection. Blue colouring of the cells shows successful intracellular uptake of β-galactosidase.

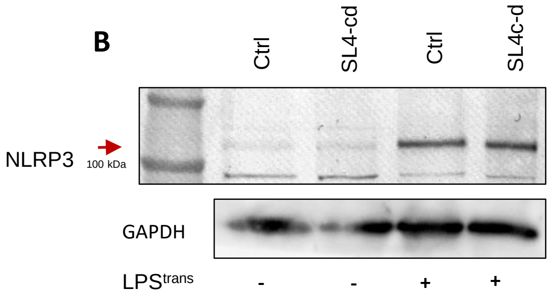

Supplemental Figure 8 (B). BDMDs of wild type mice were transfected with SL4c-d before LPS transfection. Western blot analysis show not difference in expression of NLRP3 at about 110 kDa.

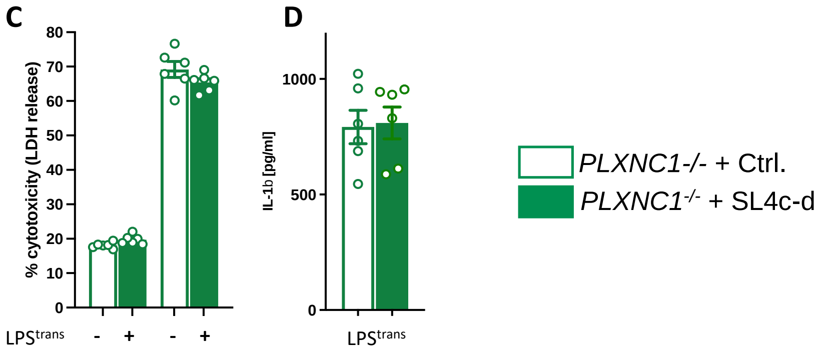

Supplemental Figure 8 (C) and (D): BDMDs of PLXNC1-/- mice were pre-treated with SL4c-d before LPS transfection. There were no significant differences in levels of (B) cytotxicity represented by LDH release, and (C) IL-1β in cell culture supernatant between control samples and samples treated with SL4c-d.

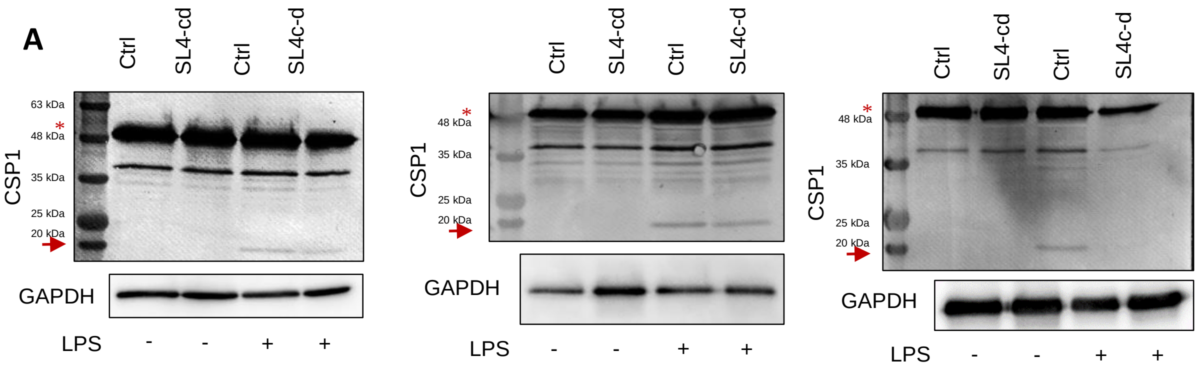

Supplemental Figure 9: (A) Western blot analysis of caspase-1 (corresponding to Fig. 5E) for three independent experimentsfor the full-form of caspase-1 at around 45/50 kDa (asterisk), and the cleaved p20 fragment (arrow) at around 20 kDa.

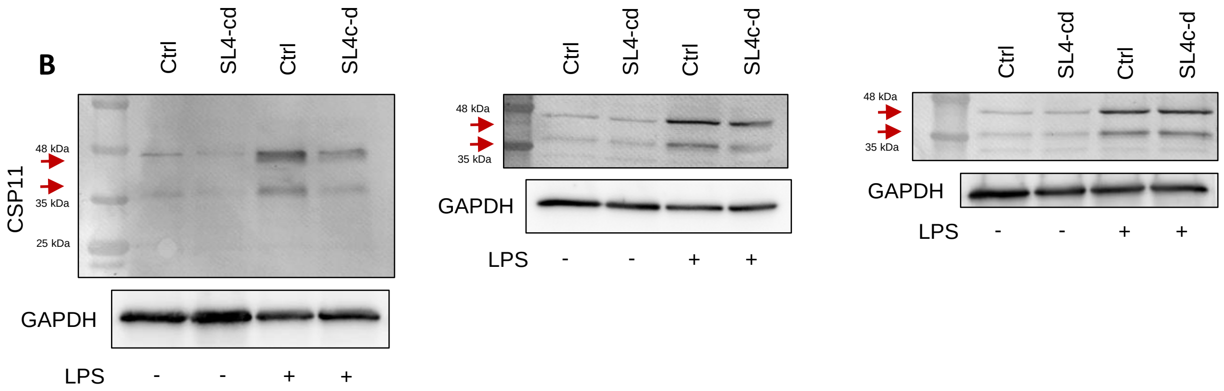

Suppl. Figure 9: (B) Western b**l**ot analysis of caspase-11 (corresponding to Fig. 5F) for three independent experiments, and densitometry the protein appearing at around (E) 45 kDa and (F) 38 kDa as indicated by the antibody manufacturer.

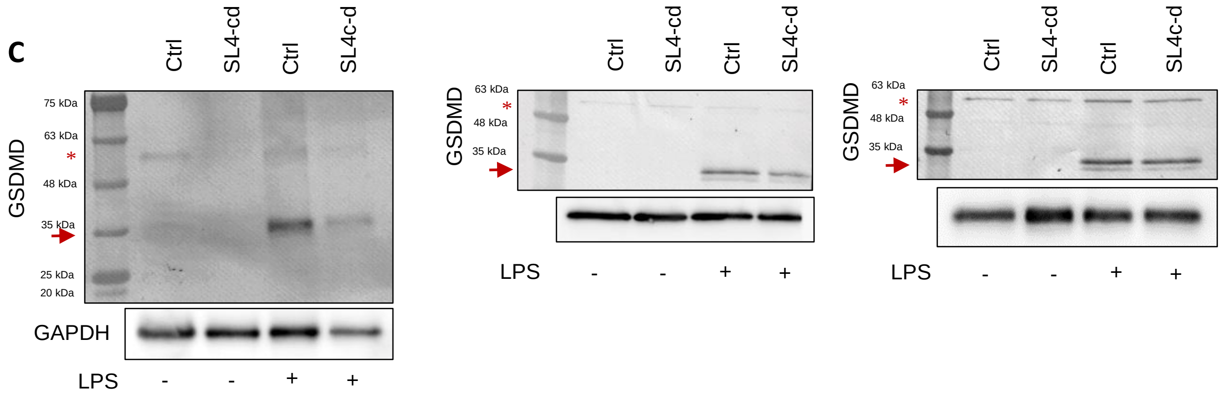

Suppl. Figure 9: (C) Western blot analysis of GSDMD (corresponding to Fig. 5G) for three independent experiments, the full sized form at around 50 kDa (asterisk), and the cleaved N-terminal fragment at around 30 kDa (arrow).

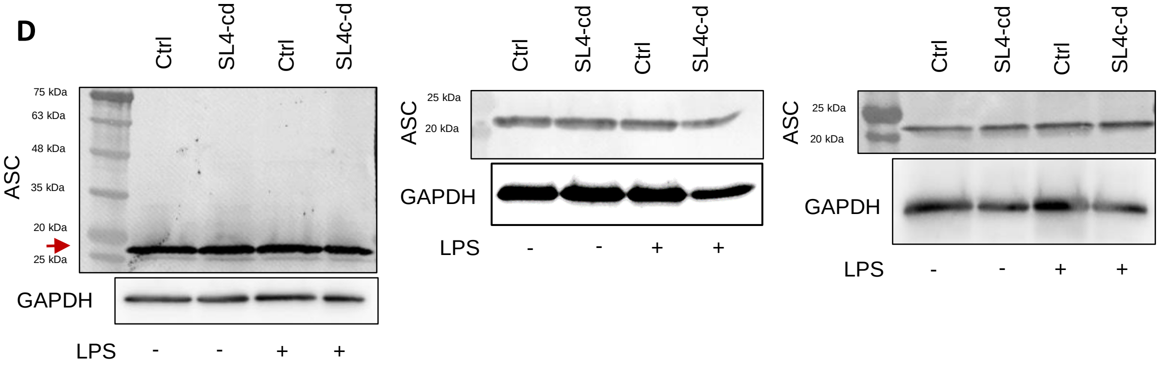

Suppl. Figure 9: (D) Western blot analysis of ASC (corresponding to Fig. 5H) for three independent experiments

D

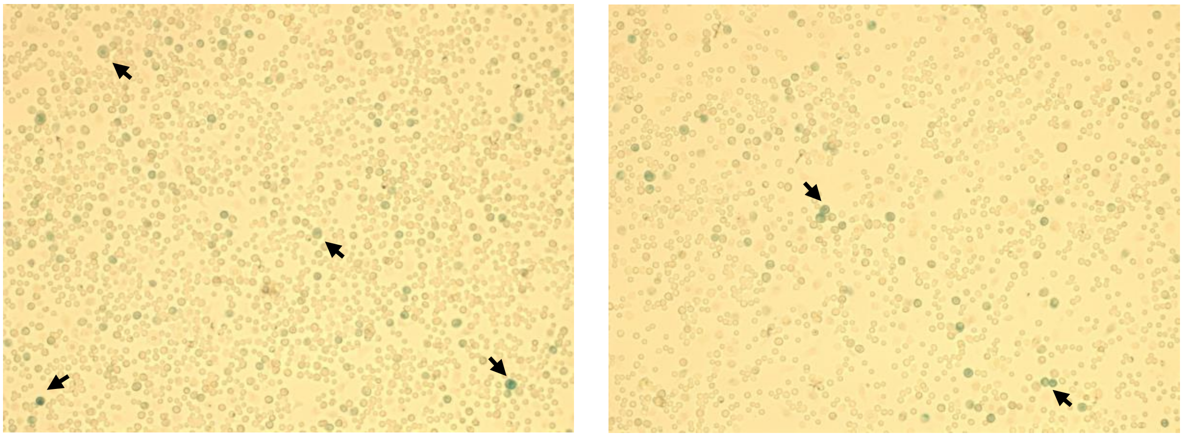

Supplemental Figure 10. Micoscopy of peritoneal lavage of wild type mice 24 hours after i.v. injection of β-galactosidase coupled to Arg9 and respective staining. Arrows point to exemplary cells showing β-galactosidase uptake (dark blue colouring).
