# Supplementary material for: Plexin C1 influences immune response to intracellular LPS and survival in murine sepsis
Source: J Biomed Sci. 2024 Aug 21;31:82. doi: 10.1186/s12929-024-01074-x (PMC11337750; doi:10.1186/s12929-024-01074-x)
Supplement: Supplementary file 2 — Additional file 2. [file 12929_2024_1074_MOESM2_ESM.docx]

**Supplemental: Material and Methods**

**Intraperitoneal LPS injection *in vivo*:** Animal experiments were performed in accordance with the German Animal Welfare Law and approved by the appropriate local authorities (i.e., Regierungspräsidium Tübingen). The model of LPS-induced peritonitis was established by intraperitoneal injection of LPS from *E. coli* subtype O111:B4 (Sigma‒Aldrich, L4392-1MG, St. Louis, Missouri, USA) at a concentration of 40 mg/kg. During survival experiments, animals were monitored for a maximum of 96 hours; overall survival was analyzed with the Kaplan‒Meier method.

***Ex vivo* stimulation using lipid A:** BMDMs from *PLXNC1^-/-^* and wild-type mice were primed overnight with 50 ng/ml LPS from *E. coli* subtype O111:B4 (Sigma‒Aldrich, L4392-1MG, St. Louis, Missouri, USA), and then transfected with lipid A (InvivoGen, tlrl-mpls, San Diego, California, USA) at a total concentration of 2 µg/ml in the presence of Lipofectamine 2000© (3 µl/ml; Thermo Fisher, #11668019, Waltham, Massachusetts, USA) according to the manufacturer's instructions. After 6 hours, supernatants were collected for measurement of LDH activity and IL-1β.

***Ex vivo* stimulation of the NLRP3 and NLRC4 inflammasome:** For activation of the NLRP3 inflammasome, BMDMs of *PLXNC1^-/-^* and wild-type mice were primed with 100 ng/ml LPS from *E. coli* subtype O111:B4 (Sigma‒Aldrich, L4392-1MG, St. Louis, Missouri, USA) for 4 hours. Samples were then stimulated with nigericin ((InvivoGen, tlrl-nig, San Diego, California, USA) for 2 hours. Supernatants were then collected for measurement of LDH activity and IL-1β.

For activation of the NLRC4 inflammasome, BMDMs were primed for 4 hours with 600 ng/ml LPS from *E. coli* subtype O111:B4 (Sigma‒Aldrich, L4392-1MG, St. Louis, Missouri, USA) for 4 hours, and then transfected with flagellin (InvivoGen, tlrl-flic-10, San Diego, California, USA) at a concentration of 100 ng/ml using Lipofectamine 2000© (Thermo Fisher, #11668019, Waltham, Massachusetts, USA) as described above. After 5 hours, supernatants were collected for measurement of LDH activity and IL-1β.

***Ex vivo* blocking of ADCY4:** BMDMs of *PLXNC1^-/-^* mice and littermate controls were transfected with LPS as described in the main body of the manuscript. In parallel, cells were treated with an ADCY4 blocking peptide (antibodies.com, A56072, Cambridge, United Kingdom) at a concentration of 5 µg/ml.

**Transfection of β-galactosidase *ex vivo* and staining:** To ensure intracellular protein uptake when using the Xfect® Protein Transfection Kit (Takara, Kusatsu, Japan) *ex vivo*, we used – as recommended by Takara – the Beta-Galactosidase Staining Kit by the same manufacturer. Along with the SL4c-d transfection, we transfected one sample with the β-galactosidase control, following the Xfect® Transfection method (as described in the main manuscript). Afterwards, respective samples were stained with the Beta-Galactosidase Staining Kit (Takara, Kusatsu, Japan) according to the manufacturer’s instruction. Controls were then examined using a Leica DM5000B microscope (Leica Microsystems, Wetzlar, Germany).

**Transfection of β-galactosidase *in vivo* and staining:** To ensure intracellular protein uptake *in vivo* when the protein is coupled to Arg9, 40 µg β-galactosidase were mixed with 5 µg Arg9 (Genaxxon, P2286.9505, Ulm, Germany) per mouse. Before i.v. injection, the total volume was adjusted to a maximum of 5 ml/kg using sodium chloride. Respective mice received an i.v. injection into the tail vein and were sacrificed after twenty-four hours to obtain peritoneal lavage. Samples were stained and examined using the Beta-Galactosidase Staining Kit (Takara, Kusatsu, Japan) as described above.
